# MTOR-DRIVEN ALTERATIONS IN THE BRAIN MICROVASCULAR PROTEOME IN ALZHEIMER’S DISEASE

**DOI:** 10.1093/geroni/igz038.356

**Published:** 2019-11-08

**Authors:** Andy Banh, Carlos Pomilio, Stacy A Hussong, Veronica Galvan

**Affiliations:** 1 Barshop Institute of Longevity and Aging Studies - UT Health San Antonio, San Antonio, Texas, United States; 2 University of Buenos Aires, Buenos Aires, Argentina, Argentina; 3 University of Texas Health Science Center at San Antonio, San Antonio, Texas, United States; 4 Barshop Institute for Longevity and Aging Studies, San Antonio, Texas, United States

## Abstract

Therapeutic interventions for Alzheimer’s disease (AD) remain limited due to an incomplete understanding of the molecular mechanisms of its onset and progression. Cerebrovascular dysfunction occurs early during disease development. Attenuation of mTOR, a key regulator of aging, attenuates and reverses cerebrovascular deficits by restoring cerebral blood flow, brain vascular density, neurovascular coupling, and vascular amyloid-β clearance in hAPP(J20) mice expressing human amyloid precursor protein carrying two FAD-associated mutations. The mechanisms by which mTOR attenuation alleviates AD pathology are poorly understood. This study defined changes in the microvascular proteome of hAPP(J20) mice arising from mTOR attenuation. At 7 months of age, hAPP(J20) mice were fed vehicle- or rapamycin-supplemented diet (2.24 mg/kg/day) for 4 months. Mass spectrometry of collected brain microvasculature identified significant changes in 840 of 3361 proteins (p<0.05). mTOR attenuation led to significant changes in 26 of these proteins, some of which are involved in pathways including tight junction regulation, calcium signaling, and actin cytoskeleton regulation. Candidate mediators of mTOR-driven cerebrovascular dysfunction were identified by selecting proteins that were aberrantly altered in hAPP(J20) microvasculature and normalized by rapamycin. Examples include members of the heterogeneous nuclear ribonucleoprotein family (hnRNPA/B, hnRNPD) which regulate the mRNA stability of genes related to cellular cycle arrest and inflammatory cytokines as well as localization of crucial mRNA involved in nitric oxide signaling. Also included are nucleoporin 54 and vacuolar ATPase assembly factor, both of which are altered in aging and neurodegeneration. Subsequent studies will elucidate the role of these proteins in mTOR-driven cerebrovascular dysfunction in AD.

